# Assessment of online self-testing and self-sampling service providers for sexually transmitted infections against national standards in the UK in 2020

**DOI:** 10.1136/sextrans-2021-055318

**Published:** 2022-04-12

**Authors:** Eleanor Clarke, Paddy J Horner, Peter Muir, Katy M E Turner, Emma Michele Harding-Esch

**Affiliations:** 1 Clinical Research Department, London School of Hygiene & Tropical Medicine, London, UK; 2 NIHR Health Protection Research Unit in Behavioural Science and Evaluation, School of Population Health Sciences, University of Bristol, Bristol, UK; 3 Unity Sexual Health, University Hospitals Bristol and Weston NHS Foundation Trust, Bristol, UK; 4 South West Regional Laboratory, National Infection Service, United Kingdom Health Security Agency, Bristol, UK

**Keywords:** testing, antimicrobial resistance, sexual health, public health

## Abstract

**Objectives:**

Online testing for STIs may help overcome barriers of traditional face-to-face testing, such as stigma and inconvenience. However, regulation of these online tests is lacking, and the quality of services is variable, with potential short-term and long-term personal, clinical and public health implications. This study aimed to evaluate online self-testing and self-sampling service providers in the UK against national standards.

**Methods:**

Providers of online STI tests (self-sampling and self-testing) in the UK were identified by an internet search of Google and Amazon (June 2020). Website information on tests and associated services was collected and further information was requested from providers via an online survey, sent twice (July 2020, April 2021). The information obtained was compared with British Association for Sexual Health and HIV and Faculty of Sexual and Reproductive Healthcare guidelines and standards for diagnostics and STI management.

**Results:**

31 providers were identified: 13 self-test, 18 self-sample and 2 laboratories that serviced multiple providers. Seven responded to the online survey. Many conflicts with national guidelines were identified, including: lack of health promotion information, lack of sexual history taking, use of tests licensed for professional-use only marketed for self-testing, inappropriate infections tested for, incorrect specimen type used and lack of advice for postdiagnosis management.

**Conclusions:**

Very few online providers met the national STI management standards assessed, and there is concern that this will also be the case for service provision aspects that were not covered by this study. For-profit providers were the least compliant, with concerning implications for patient care and public health. Regulatory change is urgently needed to ensure that all online providers are compliant with national guidelines to ensure high-quality patient care, and providers are held to account if non-compliant.

## Introduction

STIs are an increasing public health problem in the world,^1^ including the UK.^2^ Early diagnosis is a core intervention for guiding appropriate management, thus reducing the risk of antimicrobial resistance (AMR) emergence, preventing sexual and reproductive health (SRH) sequelae and reducing onward transmission.^1^ Therefore, access to validated and approved testing services is vital. Tests for self-directed use available to purchase online (‘online tests’) are increasingly popular, especially due to the COVID-19 pandemic.^3^ Even pre-COVID-19, online tests were widely viewed as an asset to public health, with studies demonstrating they can overcome barriers such as stigma and inconvenience,^2 4^ and were the second most frequent testing service type in the UK’s National Chlamydia Screening Programme in 2019.^2^ Online tests come in two main forms: self-sampling, where the user can order a kit and take a specimen independently before posting for laboratory testing^5^ and self-testing, where the user collects a specimen, conducts and interprets the test themselves.^5^

However, drawbacks to online testing have been widely reported. Barriers for use include language and health or digital literacy.^4^ Lack of interaction with a health professional may also worry users and can result in improper management of infections.^6^ UK standards for providers of sexual health services are published by BASHH and the Faculty of Sexual and Reproductive Healthcare (FSRH).^7 8^ These standards stipulate that services must include health promotion and prevention interventions and correct signposting information,^7 8^ however, these may not always be present,^9^ leaving users vulnerable to making misinformed decisions. Additionally, private testing may result in under-reporting to national surveillance systems, posing issues for epidemiological monitoring.^6 10^

A key concern of online testing is the quality of the tests themselves. Although regulatory standards such as CE-marking are often used to assess quality,^11^ this may not always indicate good performance.^12^ Poor diagnostic accuracy can lead to false-positives resulting in unnecessary treatment, with AMR risk and relationship implications, and false-negatives can result in further transmission and SRH sequalae.^13^ Furthermore, some testing panels include infections that are not recommended for routine testing (eg, *Mycoplasmas* and *Ureaplasmas*).^14 15^

In this study, we assessed whether UK patients accessing online STI tests are receiving quality of care consistent with national STI diagnostics and management standards. First, we identified and characterised online test providers in the UK, before comparing them with BASHH guidelines^7 16^ and FSRH/BASHH standards.^8^

## Methods

### Internet search of providers

To identify tests available in the UK, a structured search of Google and Amazon was completed on 27 June 2020. These platforms were chosen to enable both services and purchasable products to be identified. Search details are available in online supplemental table 1. To produce results similar to what a consumer would find, searches used layman’s terms and less well-known infections (eg, trichomoniasis) were not included. Also, terms that produced mainly educational or medical results (eg, “sexually transmitted infections”) were not included.

10.1136/sextrans-2021-055318.supp1 Supplementary data

According to click through data,^17^ most Google users do not go past the first page of search results, however for thoroughness the first five pages of results were screened by title and description. All Amazon results were screened. Inclusion criteria for a provider were:

1. The test had to be available in the UK.
2. The test had to be either self-sampling or self-testing.
3. The test was not provided by an individual borough (administrative unit), as these are geographically limited.

However, services commissioned by the National Health Service (NHS) that covered more than one borough were included. This was to represent this service type, available more generally to the UK population, in our findings.

Data on tests available were extracted iteratively from eligible websites. For products identified through third-party sellers, the original provider was identified and any other tests they provided also recorded.

### Provider questionnaire

Further information was requested from providers and associated laboratories through an online questionnaire sent in July 2020, guided by categories identified during data extraction and guidelines.^7 8 16^ Questionnaires were tailored for each provider. The full set of questions is available in online supplemental table 2. In March 2021, BASHH published a position statement^12^ regarding online services, emphasising the existence of poor practice. The statement called for increased regulation of these services, to enable providers not conforming with national guidelines to be held to account. Consequently, the questionnaire was sent again in April 2021 to the providers who did not respond in 2020, in the hope that the position statement publication would increase the response rate.

### Comparison with guidelines

Data obtained from providers were categorised into: test audience, pretest process, test process (test type and specimen type), health information, postdiagnosis actions (eg, follow-up and treatment) and accreditation. Comparison of tests with BASHH and FSRH guidelines was then conducted.^7 8 16^ Not all standards or all aspects of each standard could be measured (eg, laboratory turnaround times or safeguarding), as they referred to internal processes or information that was not available on public-facing websites. Accessibility (eg, languages) was not considered to be within the scope of this work. A full list of pathogen-specific guidelines is available alongside online supplemental table 3.

## Results

### Overview of provider responses

The Google and Amazon search returned 13 self-test and 18 self-sample providers, as well as two laboratories that serviced multiple providers. All of the self-test providers and 13 of the self-sampling providers were private. All but one self-sample providers were from the UK, self-test providers were global but available on UK platforms. In the first round of surveys, two providers completed the questionnaire, and one requested a phone call. The second round prompted four more replies. Therefore, most information was collected from provider websites. Provider names have been anonymised, in accordance with the survey terms of consent (online supplemental table 2). Guidelines that providers were compared with are summarised in table 1. Tests and specimen types are shown in table 2, with comparison to national guidelines. Further test details are in online supplemental table 3A, B. Overall, providers closest to the guidelines were NHS-commissioned free services, providing an appropriate range of tests, correct sample types and comprehensive information.

**Table 1:** Summary of recommendations from the BASHH standards for the management of STIs^7^ and the joint BASHH/Faculty of Sexual and Reproductive Healthcare (FSRH) standards for online and remote providers of sexual and reproductive health services^8^

Theme	Summary	Relevant BASHH standards^7^*	Relevant FSRH/BASHH standards^8^*
1. Test audience and pretest process	Symptomatic patients are not advised to use online services (however this guidance was modified due to COVID-19 to maintain access to services, and low-risk symptomatic patients could use online services).^18^ Providers of STI care should have in place an effective triage system to direct users to the most appropriate service. Services should take a medical and sexual history. Those who need HIV postexposure prophylaxis should not use online services and should be directed to a clinic.	1.2.6 1.5.7 1.5.8 1.5.9 1.5.10 2.4.5 2.4.7 2.4.8 2.5.2 2.5.8	3.1.3 3.2.3
2. Test process	Specimens for chlamydia, gonorrhoea, syphilis and HIV should be taken from all exposed sites as a minimum. Specimens for microbiological testing obtained during the examination should be in line with national guidance. All providers of services commissioned to manage STIs should use the ‘gold standard’ test for the infection they are screening for. Service users should be advised about the sensitivity of the assays for detecting infection.	2.4.5 2.4.8 2.5.11 2.5.14 2.5.15 3.4.2 3.4.4 3.4.8 3.4.10 3.5.1 3.5.6	3.3.2 3.3.3
3. Health information	Service users should be fully informed on the nature and limitations of the test, as well as have access to health promotion and prevention interventions including encouragement of safer sex behaviour and condom usage.	2.4.5 2.5.12	2.1.6 3.3.3
4. Postdiagnosis actions	Clear pathways with choices for individuals to obtain care, treatment and further management must be available if an STI is identified. Service users must be given information on the need for partner notification, re-testing as appropriate and advised that this is part of STI management. If services are unable to provide specialist support or additional tests, the provider should be able to provide onwards referral. Treatment should follow national guidelines.	2.4.5 2.4.10 4.4.1 4.4.8 4.4.11 4.4.16 4.5.5 4.5.13 8.4.6 8.4.8	1.1.12 1.1.13 3.5.6
5. Accreditation	Service providers should have relevant accreditation such as CE-marked products, United Kingdom Accreditation Services accredited laboratories and be registered with the Care Quality Commission. Services should comply with the joint FSRH/BASHH standards for online providers^8^ and other BASHH guidelines.	2.4.1 2.5.1 3.4.1 3.5.4 3.5.10 3.5.12 6.4.8	1.4.2 1.4.12 3.3.1

These were used to assess whether online providers were providing an adequate standard of care to patients.

*Not all standards or all aspects of each standard were able to be measured due to the nature of information available on provider websites.

**Table 2:** Pathogens tested for by online self-test and self-sample providers, and the samples requested for each pathogen, compared against national BASHH standards for the management of STIs^7 16^

Type	Provider ID	Chlamydia	Gonorrhoea	Oral/Rectal chlamydia or gonorrhoea	Syphilis	HIV	Hepatitis B	Hepatitis C	Herpes (1 and/or 2)	Trichomoniasis	*Mycoplasma genitalium*	*Mycoplasma hominis*	*Ureaplasmas*	*Gardnerella (as indicator of bacterial vaginosis*)	*Haemophilus ducreyi*	Human papillomavirus	Yeasts
BASHH recommendation		V (female), U (male), C if clinician collected	V (female), U (male)	S	B or lesion swab	B	B	B	Lesion swab or B	V, US, U	U (male and female), V/C (female)— symptomatic only	Not advised for routine testing	Not advised for routine testing	Not recommended as an indicator of bacterial vaginosis	Lesion swab— symptomatic only	Lesion swab—symptomatic only (screening uses C which are clinician collected)	V— symptomatic only
Self-test	1					B											
	2	U/C/US	C/US		B					V							
	3	U/C/US	C/US		B	B	B	B									
	4	C															
	5	S	S						B	V							
	6	U/C/US	?		B	B/O	B	B									
	7									V							
	8	?	S		B				B								
	9	V															
	10	C															
	11					B											
	12					B											
	13									?				?			?
Self-sample	14	U/V	U/V	S	B	B	B	B	U/V/S	U/V	U/V (Sp?)		U/V	U/V		V	
	15	U/V	U/V	S	B	B	B	B	U/V/S	U/V	U/V (Sp?)		U/V	U/V		V	
	16	U/V	U/V	S	B	B				U/V							
	17†	U/V	U/V	S	B	B											
	18	U/V	U/V	S	B	B	B	B									
	19†	U/V	U/V	S	B	B	B	B									
	20	U*	U*	S	B	B	?	B	B/S/U	U	U (Sp?)		U	U		V	
	21	U/V	U/V	S	B	B			?	?	?	?	?		?	V	
	22	U	U		?	?	B	B	B	U	?		?	?		V	
	23	U/V	U/V	S	B/S	B	B	B	B/S/U	U/V	U/V		U/V	U/V		V	V
	24	U	U	S	B	B	B	B	B/U	U	U (Sp?)		U	U			
	25†	U/V	U/V	S	B	B											
	26†	U/V	U/V	S	B	B											
	27	U/V	U/V		B	B	B	B	U/V	U/V	U/V (Sp?)		U/V	U/V			
	28	U	U		B	B	B	B	B	?	?		?	?		V	
	29	U	U														
	30	U/V	U/V	S	B	B	B	B	U/V	U/V	U/V		U/V	U/V			
	31†	U/V	U/V	S	B	B	B	B									

A full list of guidelines compared against is available in online supplemental table 3.

This reflects where a provider explicitly states what sample is used. This is often not included on package tests, so may not reflect all tests offered.

*Most tests requested urine, but a vaginal swab was available separately.

†Indicates a free service that is commissioned by the National Health Service.

?, specimen unclear; B, blood; O, oral transudate; S, swab; Sp?, species unclear; U, urine; US, urethral swab; V, vaginal swab.

### Test audience and pretest processes

Theme 1 was often not met. Although low-risk symptomatic patients were eligible to use online services at the time of data collection due to COVID-19 modifications to maintain access to testing,^18^ private self-sample providers who advertised to symptomatic patients did not distinguish between severity of symptoms, and testing for individuals with severe symptoms, including pelvic pain, was recommended. Advice on accessing HIV postexposure prophylaxis (PEP) was not mentioned by eight of the self-sample providers (seven private, one NHS-commissioned). With regard to triage or history taking pretesting, some self-sample providers (both private and NHS-commissioned) used an online questionnaire (the contents of which were not analysed) to recommend tests, but most providers did not have this feature.

Self-test providers did not appear to provide any form of triage as websites seemed primarily commercial and test inserts were mostly unavailable. However, five self-test providers offered tests that were marked as professional-use only.

### Test process

While both types of providers did offer tests for the minimum requirement in theme 2, these were often available individually or in various packages, leaving users able to pick and choose. For self-testing kits, the main pathogens were chlamydia (n=8), HIV (n=5) and gonorrhoea (n=5). Less common were herpes (n=2), trichomoniasis (n=4), syphilis (n=4), hepatitis B (n=2), hepatitis C (n=2), *Gardnerella* (n=1) and *Candida albicans* (n=1).

All self-sample providers offered tests for chlamydia and gonorrhoea, but availability varied for other tests (table 2). Self-sample tests were available in various combinations, some with 12 tests in one bundle. Free services provided a smaller range of tests than paid-for services. Multiple private self-sample services offered tests individually or within bundles for organisms generally regarded as commensal, such as *Ureaplasmas* or *Mycoplasmas* but species was sometimes unclear. Private self-sample providers sometimes exaggerated the importance of testing for these commensal organisms when compared with the literature.^14 15^ *Gardnerella* infection was repeatedly used as a proxy for bacterial vaginosis, contrary to recommendations.^19^ Additionally, two paid self-sample services claimed an advantage over the NHS by testing for organisms not included in routine testing.

Specimen type often conflicted with guidelines (table 2). Self-test sample types were not assessed against these guidelines as they were developed for laboratory-based diagnostic methods, however five self-test providers requested cervical samples, which should be clinician-collected.^20^ For self-sample services, five providers (all private) requested urine samples for chlamydia and gonorrhoea; four of these had no option for the recommended vaginal swab samples.^20 21^ The remaining provider did offer a vaginal swab, however this was offered separately from their main test package. For herpes, at least seven private self-sample providers requested urine samples. BASHH guidance states ‘Urine tests are inappropriate for the diagnosis of herpes’ and instead recommends that lesion swabs are taken for Nucleic Acid Amplification Testing (NAAT), or blood for serology in certain circumstances.^22^

Eleven self-test providers reported sensitivity and specificity estimates, which were all >85%, however information about reference tests or sample sizes was often unavailable. Due to lack of website information and low survey response rate, it was not possible to obtain information on diagnostic methods and accuracy from most self-sample providers. Four private self-sample providers gave values for ‘accuracy’ over 95%, however this was not mentioned for all tests. One NHS-commissioned self-sample provider gave sensitivity and specificity of >95% for all tests offered.

### Health information and signposting

To assess theme 3, we reviewed whether sites gave information on STI symptoms, window periods, transmission routes and health promotion. It was difficult to ascertain health promotion materials for many self-test providers as package inserts were often unavailable. As some self-tests were marked as professional-use only, it is expected that information would be targeted at healthcare professionals.

Only five self-sample providers (four NHS-commissioned, one private) provided information on all topics of window periods, transmission routes, symptoms and infection prevention. For other self-sample providers (mostly private, but one NHS-commissioned), information was not on the test page or was inconsistently mentioned. One private self-sample provider gave links to Wikipedia.

### Follow-up/Treatment

It was difficult to assess whether theme 4 was met as postdiagnosis processes were not always shared. All self-test providers that did include this information advised seeing a health professional after a positive result. For self-sample providers, options included a private consultation, treatment ordered online (mainly for chlamydia) or advice to visit a general practitioner. Partner notification was often mentioned non-specifically and may instead have been discussed postdiagnosis. Exact treatment options were unclear, however one private self-sample provider offered an oral course of azithromycin and cefixime for gonorrhoea which was easy to purchase online, instead of the recommended first-line treatment of intramuscular ceftriaxone.^21^

### Accreditation

Although the standards do not refer to accreditation for self-tests, it is recommended that they hold the CE-mark.^5 8^ Eleven self-test providers had at least one of their tests CE-marked, two claimed WHO approval and one claimed Food and Drug Administration accreditation. One self-test provider marked their chlamydia and gonorrhoea self-tests with an NHS logo, describing themselves as an NHS provider, but whether that product had received NHS endorsement was unclear. For self-sample providers, United Kingdom Accreditation Service (UKAS) accreditation was claimed, however, it was often used as a blanket term for the laboratory without details of the specific service that had received accreditation.^23^ UKAS accreditation was present for the two main laboratory providers identified, however it may not have covered all tests that were being offered. Care Quality Commission accreditation was present for 12 self-sample providers (both private and NHS-commissioned), although mostly only for laboratories used, as opposed to the providers themselves.

## Discussion

This study identified and analysed 31 providers of online tests in the UK. We found significant areas of suboptimal service for both self-test and self-sample providers that often conflicted with national guidelines on STI diagnostics and management. These included a lack of health promotion information, lack of triage, use of tests licensed for professional-use only marketed for self-testing, inappropriate infections tested for and incorrect specimen type used. As a result, users are at risk of taking unnecessary tests, with poor performance, that could lead to incorrect results, inappropriate management and receiving inadequate clinical information and support.

This study had limitations. Questionnaire response rate was low, despite a follow-up in 2021 following the BASHH position statement publication,^12^ meaning that not all aspects of care could be evaluated. Data considered missing in our analysis may have been available once the user had bought the test. Furthermore, data were extracted from websites in July 2020 but providers may have subsequently updated their websites. Although our internet search was comprehensive, it is not possible to identify all online STI test providers, and these change on a regular basis in this rapidly evolving field. The sample analysed here may therefore not be fully representative of all providers. This lack of representativeness may be further compounded by the small number of providers who responded to the survey and for whom we therefore have more extensive data. However, the low response rate we observed has been seen in similar studies where providers were contacted for information.^24 25^ In addition, as our study was unfunded, tests could not be purchased to identify whether information was available postpurchase. This also meant we were unable to test the services independently, either from a user perspective through a ‘mystery shopper’ exercise, or from a diagnostic accuracy perspective by independently assessing test performance claims. We also could not assess all parts of the standards as they referred to aspects not available for public access. These are next steps for future work, as well as assessing other factors such as triage questionnaire content and accessibility, and comparing against new guidelines as they are developed.^26^ Despite this, we were able to collect large amounts of information from provider websites, giving an accurate perspective of what a consumer would experience when choosing to use an online testing service.

While it was difficult to assess test performance in the identified providers due to lack of available information and inability to perform independent evaluations, it is expected that test performance was suboptimal in at least some instances.^25^ BASHH guidelines note that chlamydia and gonorrhoea tests should be NAAT-based.^20 21^ However, many non-NAAT chlamydia and gonorrhoea self-tests were available. These tests should be ‘used with extreme caution’ due to possible poor performance.^7^ Although many products had CE-marks, as noted by BASHH,^12^ this is easily obtainable and tests may not have been adequately validated. Using incorrect sample types, or being sold tests approved for professional-use only, as seen in our evaluation, may exacerbate poor test performance and add to this issue.^20–22^

The lack of appropriate health information given by self-sample providers poses a risk to users on multiple levels. Access to healthcare professionals as part of online STI services is recognised as important for offering information, technical assistance and support.^27^ Receiving accurate information regarding appropriate services and tests is critical to providing appropriate patient care, ensuring that patients receive the correct tests relevant to their situation. In contradiction to this, we found that several online providers specifically targeted patients with severe symptoms, as well as not signposting users to vital services such as PEP for HIV.^7^ Patients were also frequently offered testing for commensal *Mycoplasmas* and *Ureaplasmas*,^14^ which could lead to unnecessary costs, treatments and results of uncertain significance,^15 28^ resulting in emotional distress and poor antimicrobial stewardship. These additional tests were only found in private services, suggesting that they may be more motivated by profit than by high-quality healthcare provision.^29^ It would be important to understand why individuals choose to pay for testing rather than opting for free services, to ensure patients are offered the best possible care.

While this is the first assessment of UK online STI testing providers to our knowledge, studies in other countries externally assessing online test providers have reported similar results. A 2010 study of online tests in America performed independent assessments of online STI test providers, finding that they were hard to contact, and although self-tests had poor performance, self-sample tests had high accuracy.^25^ Providers of chlamydia online tests in The Netherlands were found to often not meet quality indicators regarding health promotion or follow-up (especially self-tests), but the process of an evaluation taking place did provoke providers to improve their service.^24^ Similarly, an Australian study of HIV self-tests showed that none conformed to national product guidelines, and often had inadequate pretest information and linkage to care.^30^ These studies demonstrate that suboptimal online testing service provision is a problem across the world. Actions such as publications highlighting short-falls and position statements with recommendations may create short-term impacts. However, if there are no mechanisms to maintain improved practice and prevent providers from, for example, appearing under a different name,^31^ these efforts are of little long-term benefit. For there to be sustained improvements in patient care, regulatory change is needed so that providers are regularly monitored and can be held to account. Although the UK’s Medicines and Healthcare products Regulatory Agency does have a #FakeMeds campaign, this is clearly not sufficient for ensuring appropriate patient care. Services need to be frequently evaluated against national guidelines, which must also be continually updated to adapt to evolving service provision.^8 12^

## Conclusion

Online testing is a welcome addition to STI diagnostics, offering a convenient and flexible option for users. However, the proliferation of providers that do not follow guidelines, in particular for-profit sites, jeopardises these advantages and puts users at risk. If current trends continue, online testing usage will increase, resulting in more online providers as demand rises. Regulatory change is required to ensure that the standard of care received online meets national guidelines to protect patients and the wider population from the repercussions of underperforming or inappropriate tests. If we do not act now, patients will continue to receive suboptimal care with potentially significant adverse personal, clinical and public health implications.

**Key messages**

- Online providers help overcome many barriers to STI testing and are increasingly popular, but quality of services is not assured.
- Many online testing services, particularly for-profit providers, did not comply with national guidelines.
- Regulatory change is required to ensure online providers comply with national guidelines and are held to account if they do not.

## Data Availability

Data are available on reasonable request. The majority of data are included in the supplementary table. Confidentiality of providers would have to be considered, in-line with the study ethics and consent procedures, for requests of any additional data.
