# Supplementary material for: Assessment of online self-testing and self-sampling service providers for sexually transmitted infections against national standards in the UK in 2020
Source: Sex Transm Infect. 2022 Apr 12;99(1):14–20. doi: 10.1136/sextrans-2021-055318 (PMC9887362; doi:10.1136/sextrans-2021-055318)
Supplement: Supplementary data [file sextrans-2021-055318supp001.pdf]

An assessment of online self-testing and self-sampling service providers for sexually transmitted infections against national standards in the United Kingdom in 2020. – Supplementary Material

Supplementary Table 1: *Search strategy used in Google. SS = self-sample provider, ST = self-test provider. For each search (1-5) the search terms for each concept were combined with the Boolean operator AND. When the acronyms STI (sexually transmitted infection) and STD (sexually transmitted disease) were included in their full form, results were mainly educational or medical, so they were not included in the search. The results for each were listed and pooled to produce a final list of providers. Amazon search terms were similar, but searches were run individually without Boolean operators. The first 5 pages of Google were searched. The search was run on 27/06/2020.*

Supplementary Table 1

Search Number	Search Concepts (combined with AND)			Number of Providers Identified by Google
	Online	STI	Test	
1	home OR online OR instant OR rapid	sti OR std	test	20 (15 SS, 5 ST)
2	home OR online OR instant OR rapid	chlamydia OR gonorrhoea OR syphilis OR hiv OR herpes	test	16 (11 SS, 5 ST)
3	home OR online OR instant OR rapid	sti OR std	test OR diagnosis OR diagnostic	19 (15 SS, 4 ST)
4	home OR online OR instant OR rapid	chlamydia OR gonorrhoea OR syphilis OR hiv OR herpes	test OR diagnosis OR diagnostic	16 (11 SS, 5 ST)
5	home OR online OR instant OR rapid	sti OR std OR chlamydia OR gonorrhoea OR syphilis OR hiv OR herpes	test OR diagnosis OR diagnostic	13 (11 SS, 2 ST)

Clarke E, *et al*. *Sex Transm Infect* 2023; 99:14–20. doi: 10.1136/sextrans-2021-055318

An assessment of online self-testing and self-sampling service providers for sexually transmitted infections against national standards in the United Kingdom in 2020. – Supplementary Material

<u>Supplementary data 2 – Questionnaires</u>

Questionnaires were adapted to each provider, depending on the tests they provide and the information already available. For questions pertaining to specific test details, and where providers had more than one test, all tests were listed where the template specifies [Test Name/Pathogen]. All questionnaires included the following consent statement:

Dear Sir/Madam,

Thank you for taking part. The information collected during this survey will be used in a research project investigating the quality and availability of online tests for sexually transmitted infections (STIs), taking place at the London School of Hygiene & Tropical Medicine.

This survey is in reference to your diagnostic test [test description and website link]. By providing information, you are consenting to have that information included in this project. No identifying information such as the company name will be included in any published work, and any information collected will be securely stored by the study team. If you wish to withdraw consent at any time after completing the survey, please e-mail [e-mail address] (study lead).

The survey is separated into three parts, 1) pre-test information 2) test performance and 3) post-test information. All questions are optional.

You may also reply in email or document form to [e-mail address] if you would prefer, and do not hesitate to email any questions about the survey or the project.

2a: Self-Test Provider Questionnaire

	Question Text	Answer Options	
1	Is the [test details] suitable for someone who has symptoms of an STI?	☐Yes	
		☐No	
		☐Other (Please Specify)	
2	Is the [test details] suitable for home use by someone who is not a clinical professional?	☐Yes	
		☐No	
3	What window period (time between being infected and the infection being detectable by the test) do you advise before using the [test detail]		Window Period (days)
		[Test Name/Pathogen]	*Free text*
4	What accreditation does your test have (e.g. CE mark, ISO)?	*Free text*	

An assessment of online self-testing and self-sampling service providers for sexually transmitted infections against national standards in the United Kingdom in 2020. – Supplementary Material

5	What specimen does your test require? (Tick all that apply)		Urine (male)	Urine (female)	Vaginal Swab	Cervical Swab	Blood	Urethral Swab	Other
		[Test Name/Pathogen]	☐	☐	☐	☐	☐	☐	Free text

6	Please describe the target and mechanism used for your diagnostic test		What is the diagnostic target for this pathogen in your test? e.g. antigen, antibody	What is the mechanism of this test? (e.g. lateral flow immunochromatographic assay)
		[Test Name/Pathogen]	*Free text*	*Free text*

7	Please provide information on the specificity and sensitivity of your test and how it was calculated. For reference: Sensitivity = true positives/true positives + false negatives Specificity = true negatives/true negatives + false positives If you do not have access to this information please discuss it with someone who does, or provide their details and we can contact them directly. If it is easier to provide this in document form, please email it to [student email address]		Sample Type	Sensitivity (%)	Sensitivity Calculation	Specificity (%)	Specificity Calculation	Reference Test
		[Test Name/Pathogen]	*Free text*	*Free text*	*Free text*	*Free text*	*Free text*	*Free text*

8	Please provide details of any publications or other literature describing the test's use and/or performance:	*Free text*
9	What advice is given to the user after a negative test result?	*Free text*
10	If a test is positive, what advice is given to the user for the next stage of care? (e.g. confirmatory tests, treatment options, follow up with medical professional)	*Free text*
11	If a diagnosis is positive, what advice is given in regard to partner notification	*Free text*

An assessment of online self-testing and self-sampling service providers for sexually transmitted infections against national standards in the United Kingdom in 2020. – Supplementary Material

	(including look back period)?	
12	Is any other advice is given after a positive test result?	*Free text*
13	Is there any other information you would like to include?	*Free text*

2b: Self-Sample Provider Questionnaire

<table>
<tr><td></td><td>Question Text</td><td colspan="6">Answer Options</td></tr>
<tr><td rowspan="3">1</td><td rowspan="3">Are any checks of eligibility done before providing a test to ensure the test is appropriate to the user (e.g. a patient questionnaire relating to symptoms, risk behaviours or previous diagnoses)?</td><td colspan="6">☐Yes</td></tr>
<tr><td colspan="6">☐No</td></tr>
<tr><td colspan="6">If yes, please provide details:
Free text</td></tr>
<tr><td rowspan="2">2</td><td rowspan="2">Is your service suitable for users that have symptoms of an STI?</td><td colspan="6">☐Yes</td></tr>
<tr><td colspan="6">☐No</td></tr>
<tr><td>3</td><td>What national guidelines do you follow for running your service (e.g. BASHH)?</td><td colspan="6">Free text</td></tr>
<tr><td rowspan="2">4</td><td rowspan="2">What window period (time between being infected and the infection being detectable by the test) do you advise before using the [test detail]</td><td></td><td colspan="5">Window Period (days)</td></tr>
<tr><td>[Test Name/Pathogen]</td><td colspan="5">Free text</td></tr>
<tr><td>5</td><td>What laboratory do you use for your diagnostic tests and what accreditations do they have (e.g. UKAS, ISO)?</td><td colspan="6">Free text</td></tr>
<tr><td rowspan="2">6</td><td rowspan="2">Which of the following species of Mycoplasma and Ureaplasma do you test for?</td><td>Mycoplasma hominis</td><td>Mycoplasma genitalium</td><td>Ureaplasma parvum</td><td>Ureaplasma urealyticum</td><td>Ureaplasma (not species specific)</td><td>Other</td></tr>
<tr><td>☐</td><td>☐</td><td>☐</td><td>☐</td><td>☐</td><td>Free text</td></tr>
<tr><td rowspan="2">7</td><td rowspan="2">Do any of your tests include antimicrobial</td><td colspan="6">☐ Yes</td></tr>
<tr><td colspan="6">☐ No</td></tr>
</table>

Clarke E, *et al*. *Sex Transm Infect* 2023; 99:14–20. doi: 10.1136/sextrans-2021-055318

An assessment of online self-testing and self-sampling service providers for sexually transmitted infections against national standards in the United Kingdom in 2020. – Supplementary Material

<table>
<tr><td></td><td>susceptibility testing?</td><td colspan="10">If yes, please provide details: Free text</td></tr>
<tr><td rowspan="2">8</td><td rowspan="2">What samples do you use to test for the following pathogens in the [test package name]? Tick all that apply.</td><td></td><td>Urine (male)</td><td>Urine (female)</td><td>Oral Swab</td><td>Rectal Swab</td><td>Vaginal Swab</td><td>Cervical Swab</td><td>Blood</td><td>Urethral Swab</td><td>Other</td></tr>
<tr><td>[Test Name/Pathogen]</td><td>☐</td><td>☐</td><td>☐</td><td>☐</td><td>☐</td><td>☐</td><td>☐</td><td>☐</td><td>Free text</td></tr>
<tr><td rowspan="2">9</td><td rowspan="2">Please describe the target and platform/assay used for your diagnostic tests. If any tests are done in combination, or if there are multiple tests used, please include that in the details. If you do not have access to this information, please discuss it with someone who does or provide their details and we can contact them directly. For HIV, please also specify the generation of test if applicable.</td><td></td><td colspan="5">What is the diagnostic target for this pathogen? e.g. antibody, nucleic acid</td><td colspan="4">What platform or assay is used to run this test?</td></tr>
<tr><td>[Test Name/Pathogen]</td><td colspan="5">Free text</td><td colspan="4">Free text</td></tr>
<tr><td rowspan="2">10</td><td rowspan="2">Please provide information on the specificity and sensitivity of your test and how it was calculated. For reference: Sensitivity = true positives/true positives + false negatives Specificity = true negatives/true negatives + false positives If you do not have access to this information please discuss it with someone who does, or provide their details and we can contact them directly. If it is easier to provide this in document form, please email it to [student email address]</td><td></td><td>Sample Type</td><td colspan="2">Sensitivity (%)</td><td colspan="2">Sensitivity Calculation</td><td>Specificity (%)</td><td>Specificity Calculation</td><td colspan="2">Reference Test</td></tr>
<tr><td>[Test Name/Pathogen]</td><td>Free text</td><td colspan="2">Free text</td><td colspan="2">Free text</td><td>Free text</td><td>Free text</td><td colspan="2">Free text</td></tr>
</table>

5

An assessment of online self-testing and self-sampling service providers for sexually transmitted infections against national standards in the United Kingdom in 2020. – Supplementary Material

11	Please provide details of any publications or other literature describing the test's use and/or performance:	*Free text*		
12	What advice is given to the user after a negative test result?	*Free text*		
13	What is the next stage of care when the user tests positive for the infections listed below (e.g. treatment is provided online, user is referred to a sexual health clinic, confirmatory testing is required), and what advice about partner notification is given (including look back period)?		Next stage of care	Advice/recommendations about partner notification
		[Test Name/Pathogen]	*Free text*	*Free text*
14	Do you recommend any tests be repeated at a later date?	☐ Yes		
		☐ No		
		If yes, please provide details of what recommendations are made: *Free text*		
15	If a chlamydia test is positive, what is your advice to the user regarding testing for lymphogranuloma venereum (LGV)?	*Free text*		
16	Please describe any other advice given after a positive diagnosis from any of your tests:	*Free text*		
17	Do you report your results to any external body (e.g. Public Health England) for surveillance purposes?	☐ Yes		
		☐ No		
		If yes, please provide details: *Free text*		
18	Is there any other information you would like to include?	*Free text*		

Clarke E, *et al. Sex Transm Infect* 2023; 99:14–20. doi: 10.1136/sextrans-2021-055318

An assessment of online self-testing and self-sampling service providers for sexually transmitted infections against national standards in the United Kingdom in 2020. – Supplementary Material

Supplementary Tables 3a and 3b

Providers were compared to pathogen specific guidelines where available, and other literature where guidelines have not been published: chlamydia[1], gonorrhoea,[2] syphilis,[3] HIV,[4] hepatitis,[5] herpes,[6] trichomoniasis,[7] Mycoplasma genitalium,[8] Mycoplasma hominis,[9,10] Ureaplasmas,[9,10] Gardnerella,[11] chancroid,[12] human papillomavirus,[13,14] yeasts.[15].

Supplementary Table 3a: *Description of the characteristics of self-test kits for sexually transmitted infections found available online. All products were paid for. In total, 9 providers were identified by Amazon search and 4 were identified by both Google and Amazon search.*

Provider	Source	Infection	Sample Type Requested	Positive Diagnosis Advice	Professional Use Only?	Accreditation	Sensitivity (sample type)	Specificity (sample type)	Reference Test
1*	B	HIV	Blood	Consult a doctor for confirmatory testing	No	CE, WHO approved	100% (Blood)	99.8% (Blood)	PCR
2*	A	Chlamydia	Cervical/urethral swab/Urine	Seek confirmatory testing and treatment for you and your partner	Yes	CE	91.3% (Swab/Urine)	98.1% (Swab/Urine)	PCR
		Gonorrhoea	Cervical/urethral swab		Yes	CE	97% (Swab)	96% (Swab)	Culture
		Trichomoniasis	Vaginal		Yes	CE	85.7% (Swab)	97.5% (Swab)	Another rapid test (Unnamed)
		Syphilis	Blood		Yes	CE	99.7% (Blood)	>99.9% (Blood)	TPPA
3	B	Chlamydia	Cervical/urethral swab/urine	/	Yes	CE	90% (Cervical Swab)<br>80.9% (Male Urethral Swab)<br>92.3% (Male Urine)	96.5% (Cervical Swab)<br>94.3% (Male Urethral Swab)<br>>99.9% (Male Urine)	PCR
		Syphilis	Blood		Yes	CE	>99.9% (Whole Blood)	99.7% (Whole Blood)	/
		Gonorrhoea	Cervical/urethral swab		Yes	CE	90.9% (Cervical Swab)<br>90% (Male Urethral Swab)	96.4% (Cervical Swab)<br>96.8% (Male Urethral Swab)	Culture
		Hepatitis B Surface Antigen	Blood		Yes	/	>99.9% (Whole Blood)	99.3% (Whole Blood)	/
		Hepatitis C	Blood		/	/	99.1% (Whole Blood)	99.5% (Whole Blood)	/

An assessment of online self-testing and self-sampling service providers for sexually transmitted infections against national standards in the United Kingdom in 2020. – Supplementary Material

Provider	Source	Infection	Sample Type Requested	Positive Diagnosis Advice	Professional Use Only?	Accreditation	Sensitivity (sample type)	Specificity (sample type)	Reference Test
		HIV Antigen/antibody	Blood		Yes	/	>99.9%	99.5%	/
4	B	Chlamydia	Cervical Swab (advertised as vaginal)	See a health professional	No	CE	85.7% (Cervical Swab)	98.3% (Cervical Swab)	PCR
5	A	Chlamydia	Swab (source unclear)	See a health professional	Yes	CE, FDA	98.5% "accurate"		/
		Gonorrhoea	Swab (source unclear)		Yes	CE, FDA	98.5% "accurate"		/
		Genital Herpes (HSV2)	Blood		Yes	CE, FDA	99% "accurate"		/
		Oral Herpes (HSV1)	Blood		Yes	CE, FDA	99% "accurate"		/
		Trichomoniasis	Vaginal swab		Yes	CE, FDA	98.5% "accurate"		/
6	A	Chlamydia	Cervical/urethral swab/urine	/	Yes	CE	/	/	/
		Gonorrhoea	/		Yes	CE	/	/	/
		Syphilis	Blood		Yes	CE	/	/	/
		HIV (Blood)	Blood		Yes	WHO Prequalified	/	/	/
		HIV (Oral)	Oral transudate		Yes	/	/	/	/
		Hepatitis B Surface Antibody	Blood		Yes	/	97.30%	99.20%	/
		Hepatitis B Surface Antigen	Blood		Yes	/	/	/	/
		Hepatitis C	Blood		Yes	/	99%	99.80%	/
7*	A	Trichomoniasis	Vaginal swab	See a health professional	No	CE	99%	100%	Culture
8	A	Chlamydia	/	/	No	CE	/	/	/
		Syphilis	Blood		No	/	/	/	/

An assessment of online self-testing and self-sampling service providers for sexually transmitted infections against national standards in the United Kingdom in 2020. – Supplementary Material

Provider	Source	Infection	Sample Type Requested	Positive Diagnosis Advice	Professional Use Only?	Accreditation	Sensitivity (sample type)	Specificity (sample type)	Reference Test
		Gonorrhoea	Swab (source unclear)		Yes	CE	/	/	/
		HSV1	Blood		Yes	CE	/	/	/
		HSV2	Blood		Yes	CE	/	/	/
9	A	Chlamydia	Vaginal swab	/	No	/	98.3%	/	/
10*	A	Chlamydia	Cervical Swab (advertised as vaginal)	See a health professional	No	CE	85.7 % (Cervical Swab)	98.3 % (Cervical Swab)	PCR
11	A	HIV	Blood	See a health professional for confirmatory tests	No	CE	99.6% (Blood)	/	/
12	A	HIV	Blood	See a health professional for confirmatory tests	No	CE	99.7% (Blood)	99.9% (Blood)	Enzyme immunoassa y and western blot
13	B	Gardnerella	/	/	No	/	98.5%	98.6%	PCR
		Trichomonia sis	/		No	/	100%	99%	Wet mount microscopy and culture
		Candida Albicans	/		No	/	95.5%	98.4%	Wet mount microscopy and culture

*/ Indicates the information was not stated or unclear. HSV = herpes simplex virus, TPPA = Treponema pallidum particle agglutination assay, FDA = food and drug administration, WHO = World Health Organisation. * Indicates the provider responded to the survey. Source indicates Amazon (A), Google (G) or both (B). Accreditation may have been present but the documents not immediately available to us*

An assessment of online self-testing and self-sampling service providers for sexually transmitted infections against national standards in the United Kingdom in 2020. – Supplementary Material

Supplementary Table 3b: *Description of the characteristics of self-sample provider websites and services. In total, 17 providers were identified by Google search and one was identified by both Google and Amazon search. All free providers were commissioned by the National Health Service*

Provider	Source	Window Period Stated	Advised for symptomatic users?	Pre-test Screen	Symptom Information	Transmission Information	Health Protection Information	HIV PEP signpost	Positive Result Guidance	Accreditations	Price Range
14	G	Yes	Unclear	No	Yes	Inconsistently	Condoms mentioned on two test pages, not all	Yes	Treat online or arrange a consultation	CQC	£29-£244
15	G	Yes	Unclear	No	Yes	Inconsistently	Thorough description on one test page but not all	Yes	Treat online or arrange a consultation	CQC	£27.99-£225.99
16	G	Yes	No	Yes	Yes, not on the test page	Yes, not on the test page	Yes, not on the test page	Yes	Treat online or referral	Unclear	£27.99-£99.95
17*	G	Yes	No	Yes	Yes	Yes	Yes	Yes	Refer to treatment	CQC, Claims a UKAS Accredited Laboratory	Free
18	G	Yes	No	Yes	Yes	Yes	Yes	Yes	Treat online/referral	Unclear	£28-£128
19	G	Yes	No	Yes	Yes	Yes	Yes	Yes	Treat online/referral	CQC, UKAS accredited laboratory	Free
20	G	Yes	Unclear	No	Yes, not on the test page	Yes, not on the test page	Condoms mentioned on one test page, not all	No	Treat online, consultation or referral	Unclear – UKAS badge given for quality management service, not tests provided	£35-£299
21	G	No	No	No	Yes, not for all pathogens	Yes, not for all pathogens	Links to Wikipedia	No	Signpost to local services	CQC, UKAS accredited laboratory but not accredited for all pathogens tested for	£19.99-£114.99

An assessment of online self-testing and self-sampling service providers for sexually transmitted infections against national standards in the United Kingdom in 2020. – Supplementary Material

Provider	Source	Window Period Stated	Advised for symptomatic users?	Pre-test Screen	Symptom Information	Transmission Information	Health Protection Information	HIV PEP signpost	Positive Result Guidance	Accreditations	Price Range
22	G	Yes	Yes	No	No	No	No	No	Results will be discussed with the user	Unclear	£49-£209
23	G	Yes, not for all pathogens	Yes	No	Yes, not on the test page	Yes, not on the test page and not for all pathogens	No	No	Treat online or a consultation or referral	CQC	£29.95-£299.95
24	G	Yes, not for all pathogens	Unclear	Limited	Yes, not for all pathogens and not always on the test page	Yes, not for all pathogens and not always on the test page	Condoms and safe toy use mentioned on each STI info page	No	Phone consultation	Claim they use a UKAS accredited laboratory but no further details to verify this	£95-£239
25*	G	Yes	Yes (survey), No (website)	Yes	Yes	Yes	Yes	Yes	Consultation	Unclear	Free
26	G	Yes, not for all pathogens	Unclear	No	Yes	Yes	No	No	Unclear	CQC, UKAS accredited laboratory	Free
27	B	No	Unclear	No	Yes, not for all pathogens	Yes, not for all pathogens	No	No	Consultation	CQC	£34-£225
28	G	Yes, not for all pathogens	Yes	No	Yes, not for all pathogens	Yes, not for all pathogens	No	No	Advised to see your doctor, states they will not diagnose or consult	CQC, claims UKAS laboratory however this lab is not accredited for all STIs. May be accredited for other services they provide	£37-£251
29	G	Yes	Unclear	No	Yes, not for all pathogens	No	Condoms advised	N/A (no HIV test)	Treat online/referral	CQC	£35

An assessment of online self-testing and self-sampling service providers for sexually transmitted infections against national standards in the United Kingdom in 2020. – Supplementary Material

Provider	Source	Window Period Stated	Advised for symptomatic users?	Pre-test Screen	Symptom Information	Transmission Information	Health Protection Information	HIV PEP signpost	Positive Result Guidance	Accreditations	Price Range
30	G	Yes	Unclear	No	Inconsistently	Yes, not for all pathogens	Condoms and safe toy usage mentioned on some pages but not all	Yes	Treat online/referral	CQC	£32-£200
31*	G	Yes	No	Yes	Yes	Yes	Yes	Yes	Treat online/referral	CQC, UKAS Accredited Laboratory	Free

*CQC = care quality commission, PEP = post-exposure prophylaxis, ISO = international organisation for standardization, UKAS = United Kingdom Accreditation Service. * indicates that the provider responded to the survey. Source indicates Amazon (A), Google (G) or both (B). Accreditation may have been present but the documents not immediately available to us*

Clarke E, *et al. Sex Transm Infect* 2023; 99:14–20. doi: 10.1136/sextrans-2021-055318

An assessment of online self-testing and self-sampling service providers for sexually transmitted infections against national standards in the United Kingdom in 2020. – Supplementary Material

References

1 Nwokolo NC, Dragovic B, Patel S, *et al.* 2015 UK national guideline for the management of infection with Chlamydia trachomatis. *Int J STD AIDS* 2015;27:251–67. doi:10.1177/0956462415615443 Available at: https://pubmed.ncbi.nlm.nih.gov/26538553/

2 Fifer H, Saunders J, Soni S, *et al.* 2018 UK national guideline for the management of infection with Neisseria gonorrhoeae. *Int J STD AIDS* 2019;31:4–15. doi:10.1177/0956462419886775 Available at: https://pubmed.ncbi.nlm.nih.gov/31870237/

3 Kingston M, French P, Higgins S, *et al.* UK national guidelines on the management of syphilis 2015. *Int J STD AIDS* 2015;27:421–46. doi:10.1177/0956462415624059 Available at: https://pubmed.ncbi.nlm.nih.gov/26721608/

4 Palfreeman A, Sullivan A, Rayment M, *et al.* British HIV Association/British Association for Sexual Health and HIV/British Infection Association adult HIV testing guidelines 2020. *HIV Med* 2020;21 Suppl 6:1–26. doi:10.1111/hiv.13015 Available at: https://pubmed.ncbi.nlm.nih.gov/33333625/

5 British Association for Sexual Health and HIV. 2017 Interim Update of the 2015 BASHH National Guidelines for the Management of Viral Hepatitides. 2017. Available at: https://www.bashhguidelines.org/media/1161/viral-hepatitides-2017-update-18-12-17.pdf (Accessed June 2021)

6 Patel R, Green J, Clarke E, *et al.* 2014 UK national guideline for the management of anogenital herpes. *Int J STD AIDS* 2015;26:763–76. doi:10.1177/0956462415580512 Available at: https://pubmed.ncbi.nlm.nih.gov/25861804/

7 Sherrard J, Ison C, Moody J, *et al.* United Kingdom National Guideline on the Management of Trichomonas vaginalis 2014. *Int J STD AIDS* 2014;25:541–9. doi:10.1177/0956462414525947 Available at: https://pubmed.ncbi.nlm.nih.gov/24616117/

8 Soni S, Horner P, Rayment M, *et al.* British Association for Sexual Health and HIV national guideline for the management of infection with Mycoplasma genitalium (2018). *Int J STD AIDS* 2019;30:938–50. doi:10.1177/0956462419825948 Available at: https://pubmed.ncbi.nlm.nih.gov/31280688/

9 Horner P, Donders G, Cusini M, *et al.* Should we be testing for urogenital Mycoplasma hominis, Ureaplasma parvum and Ureaplasma urealyticum in men and women? – a position statement from the European STI Guidelines Editorial Board. *J Eur Acad Dermatology Venereol* 2018;32:1845–51. doi:10.1111/jdv.15146 Available at: https://pubmed.ncbi.nlm.nih.gov/29924422/

10 Taylor-Robinson D, Horner P, Pallecaros A. Diagnosis of some genital-tract infections: part 2. Molecular tests and the new challenges. *Int J STD AIDS* 2020;31:198–207. doi:10.1177/0956462419890526 Available at: https://pubmed.ncbi.nlm.nih.gov/32009570/

11 British Association for Sexual Health and HIV. UK National Guideline for the Management of Bacterial Vaginosis 2012. 2012. Available at: https://www.bashhguidelines.org/media/1041/bv-2012.pdf (Accessed June 2021)

12 Lautenschlager S, Kemp M, Christensen JJ, *et al.* 2017 European guideline for the management of chancroid. *Int J STD AIDS* 2017;28:324–9.

doi:10.1177/0956462416687913 Available at: https://pubmed.ncbi.nlm.nih.gov/28081686/

13 British Association for Sexual Health and HIV. UK National Guidelines on the Management of Anogenital Warts 2015. 2015. Available at: https://www.bashhguidelines.org/media/1075/uk-national-guideline-on-warts-2015-final.pdf (Accessed June 2021)

14 Public Health England. Cervical screening: programme overview. 2021. Available at: https://www.gov.uk/guidance/cervical-screening-programme-overview (Accessed June 2021)

15 Saxon C, Edwards A, Rautemaa-Richardson R, *et al.* British Association for Sexual Health and HIV national guideline for the management of vulvovaginal candidiasis (2019). *Int J STD AIDS* 2020;31:1124–44. doi:10.1177/0956462420943034 Available at: https://pubmed.ncbi.nlm.nih.gov/32883171/
